# Proanthocyanidin B2 inhibits proliferation and induces apoptosis of osteosarcoma cells by suppressing the PI3K/AKT pathway

**DOI:** 10.1111/jcmm.15818

**Published:** 2020-09-10

**Authors:** Xinbo Wu, Haiyang Yu, Haichao Zhou, Zihua Li, Hui Huang, Fajiao Xiao, Shaochen Xu, Yunfeng Yang

**Affiliations:** ^1^ Department of Orthopedics Shanghai Tongji Hospital Tongji University School of Medicine Shanghai China

**Keywords:** apoptosis, osteosarcoma, PI3K/AKT pathway, proanthocyanidin B2, proliferation

## Abstract

Osteosarcoma (OS) is the most common primary malignant bone tumour in children and adolescents. The long‐term survival rate of OS patients is stubbornly low mainly due to the chemotherapy resistance. We therefore aimed to investigate the antitumoral effects and underlying mechanisms of proanthocyanidin B2 (PB2) on OS cells in the current study. The effect of PB2 on the proliferation and apoptosis of OS cell lines was assessed by CCK‐8, colony formation, and flow cytometry assays. The target gene and protein expression levels were measured by qRT‐PCR and Western blotting. A xenograft mouse model was established to assess the effects of PB2 on OS proliferation and apoptosis in vivo. Results from in vitro experiments showed that PB2 inhibited the proliferation and induced apoptosis of OS cells, and also increased the expression levels of apoptosis‐related proteins. Moreover, PB2 induced OS cell apoptosis through suppressing the PI3K/AKT signalling pathway. The in vivo experiments further confirmed that PB2 could inhibit OS tumour growth and induce its apoptosis. Taken together, these results suggested that PB2 inhibited the proliferation and induced apoptosis of OS cells through the suppression of the PI3K/AKT signalling pathway.

## 1 INTRODUCTION

Osteosarcoma (OS) derived from bone‐forming mesenchymal cells is the most common primary bone tumour in children and adolescents.^1^, ^2^, ^3^ Among the OS patients, more than 90% are high‐grade malignancies based on the grade of differentiation, and more than 20% have demonstrated significant metastasis lesions at the time of clinical diagnosis.^4^, ^5^ With the improvement of early diagnosis and treatment strategies, the 5‐year survival rate of OS patients has increased up to 70%.^6^ However, the long‐term prognosis remains poor for the patients with advanced OS over the past 30 years mainly due to the resistance to chemotherapeutic agents, in addition to the metastatic diseases and local or distant recurrence.^7^, ^8^ Furthermore, due to the toxicity of multi‐agent chemotherapy, OS patients often experience acute and late complications, such as myelosuppression and peripheral neuropathic pain, which seriously affects their quality of life.^9^ Therefore, there is an urgent need to explore new chemotherapeutic agents that not only kill the primary tumour but also reduce the adverse effects of chemotherapy.

Recently, natural drugs have become a popular researching focus since drug discovery is a high‐risk and high‐cost process.^10^, ^11^ Evidence has suggested that several natural drugs, for example, vincristine, taxanes and camptothecin, are effective anti‐tumour agents.^12^, ^13^, ^14^ Furthermore, some natural compounds have been demonstrated to own anti‐tumour effects on OS cell lines with few adverse influences.^15^, ^16^, ^17^ Therefore, researches on natural drugs may provide new ideas for the treatment of OS.

Proanthocyanidin B2 (PB2) is a polyphenolic compound derived from common dietary foods such as grape seed and cranberry.^18^, ^19^ Evidence has indicated that PB2 has a variety of pharmacological bioactivities, including anti‐oxidation, anti‐lipid peroxidation and anti‐inflammation.^20^, ^21^, ^22^ Specifically, it has been frequently reported that PB2 also possesses anti‐tumour properties without affecting the viability of normal cells.^23^, ^24^, ^25^, ^26^, ^27^ Feng et al^23^ reported that PB2 could suppress aerobic glycolysis and induce apoptosis of hepatocellular cells by disrupting the PKM2/HSP90/HIF‐1α axis. Engelbrecht et al^28^ revealed that PB2 could inhibit the proliferation and induce apoptosis of colon cancer cells through inactivating the PI3K/PKB signalling pathway. However, there still lack studies involving the biological activities of PB2 for the treatment of OS. Therefore, we set out to assess the potential antitumoral effects of PB2 on OS and to explore the underlying molecular mechanisms.

## 2 MATERIALS AND METHODS

### 2.1 Reagents

PB2, dimethyl sulphoxide, penicillin‐streptomycin, paraformaldehyde (PFA), Crystal Violet, chloroform, isopropyl alcohol, radioimmunoprecipitation assay buffer (RIPA buffer), polyvinylidene difluoride (PVDF) membranes and Dulbecco's Modified Eagle's Medium (DMEM) were purchased from Sigma‐Aldrich. TRIzol RNA isolation reagent, diethyl pyrocarbonate (DEPC), foetal bovine serum (FBS) and trypsin were obtained from Gibco. Z‐VAD‐FMK and PI3K activator 740 Y‐P were purchased from Selleckchem. The apoptosis detection kit was obtained from BD Biosciences. The Cell Counting Kit‐8 (CCK‐8) and the JC‐1 mitochondrial membrane potential assay kit were obtained from Yeasen Biotechnology. The reverse transcription‐polymerase chain reaction (qRT‐PCR) kit was obtained from TaKaRa Biotechnology. Cell mitochondrial isolation kit, bicinchoninic acid (BCA) protein assay reagents, sodium dodecyl sulphate polyacrylamide gel electrophoresis (SDS‐PAGE) equipment and phosphate‐buffered saline (PBS) were purchased from Beyotime. Information about primary antibodies was listed in Table 1.

**TABLE 1:** The primary antibodies used in the current study

Antibody	Species	Dilution ration	Supplier	Catalogue number
PCNA	Rbt	1:2000	PT	10205‐2‐AP
Caspase 9	M	1:1000	PT	66169‐1‐Ig
Caspase 3	Rbt	1:1000	PT	19677‐1‐AP
Bcl‐2	M	1:1000	CST	15071S
Bax	Rbt	1:1000	PT	50599‐2‐Ig
PARP	Rbt	1:1000	CST	9532S
cyto‐C	Rbt	1:1000	CST	11940S
AIF	Rbt	1:1000	CST	5318S
PI3K	Rbt	1:1000	PT	20584‐1‐AP
AKT	M	1:1000	PT	60203‐2‐Ig
p‐AKT	M	1:1000	PT	66444‐1‐Ig
β‐actin	M	1:2000	CST	3700

Abbreviations: CST, Cell Signaling Technology; M, mouse; PT, Proteintech; Rbt, rabbit.

### 2.2 Cell culture

Four OS cell lines (143B, MNNG, SJSA and MG‐63) and one normal human osteoblast cell line (hFOB1.19) were purchased from the Cell Bank of the Chinese Academy Sciences. Cells were cultured in DMEM with 10% FBS and 1% Penicillin‐Streptomycin. Cells were routinely harvested with trypsin and sub‐cultured when the cell density reached 90%. Cells in the exponential growth phase were used for the following experiments, and all experiments were performed in triplicate.

### 2.3 Cell proliferation assay

The effects of PB2 on OS cell proliferation were evaluated with CCK‐8 and colony formation assays. Cells were seeded in 96‐well culture plates at a density of 3 × 10^3^ cells/well in 100 μL complete culture medium for 24 hours. Then, the cells were cultured in DMEM or in DMEM with different concentrations of PB2 (30‐100 μmol/L) for 24, 48 or 72 hours. After the supernatant was removed, 90 μL DMEM and 10 μL CCK‐8 were added in each well and incubated for two hours at 37°C. And then, the absorbance of the control group (Ac) and the treatment group (At) was measured at 450 nm, respectively, using a Synergy H4 microplate reader (BioTek). Cell viability was calculated according to the formula: cell viability = [1 − (Ac‐At)/Ac] × 100%.

We specifically selected two OS cell lines (143B and MNNG) for the following in vitro experiments in this study. 143B and MNNG cells were collected with trypsin and five hundred cells per well were seeded in 6‐well culture plates in complete culture medium for colony formation assays. After 7 days of incubation, the cells were treated with PB2 at different concentrations of 0, 50, 70 and 90 μmol/L for another 7 days.^23^ And then, the cells were fixed with 4% PFA, stained with 0.1% Crystal Violet, and the images were captured using a digital camera. The colonies were counted, and the clone formation rate was calculated according to the formula: clone formation rate = number of clone/number of seeded cell × 100%.

### 2.4 Apoptosis analysis by flow cytometry

The effects of PB2 on OS cell apoptosis were analysed with an Annexin V‐FITC apoptosis detection kit. 143B and MNNG cells were seeded in 12‐well plates for 24 hours and then treated with PB2 at different concentrations of 0, 50, 70 and 90 μmol/L for another 48 hours. The cells were digested with trypsin and collected. Cell supernatants were centrifuged at 1000 rpm and washed twice with ice‐cold PBS. Cells were then stained with 5 μL Annexin V‐FITC and 5 μL propidium iodide (PI) for 30 minutes in dark at 4°C. Analysis of cell apoptosis was performed using a Cytomics FC500 flow cytometry (Beckman Coulter), and at least 1 × 10^4^ cells were measured for each measurement. Specifically, Annexin V‐FITC‐positive and PI‐negative cells were regarded as early apoptosis cells, while Annexin V‐FITC‐positive and PI‐positive cells were regarded as late apoptosis/secondary necrosis cells. These results were analysed by FlowJo software (version10; FlowJo LLC).

### 2.5 Mitochondrial membrane potential analysis

Disruption of mitochondrial membrane potential (MMP, ΔΨm) is a landmark event of early apoptosis. The JC‐1 mitochondrial membrane potential assay kit was used to measure the changes of MMP. 143B and MNNG cells were seeded in 6‐well plates for 24 hours and then treated with different PB2 concentrations (0, 50, 70 and 90 μmol/L) for 48 hours. Cells were harvested and stained with JC‐1 working solution for 20 minutes at 37°C, washed twice with JC‐1 staining buffer, and analysed by flow cytometry.

### 2.6 RNA extraction, reverse transcription and quantitative real‐time PCR

143B and MNNG cells were incubated in 6‐well plates and treated with PB2 at different concentrations of 0, 50, 70 and 90 μmol/L for 48 hours. Total RNA was extracted from the cells using TRIzol reagent and reverse‐transcribed into cDNA by using a reverse transcription kit. The levels of gene expression were determined by quantitative real‐time PCR (qRT‐PCR) using a 7500 real‐time PCR system (Applied Biosystems). The β‐actin was used as an internal reference to verify equal amounts of cDNA, and the levels of different genes expression were quantified using the $2^{‐\Delta \Delta C_{t}}$ method. The primers used for PCR were presented in Table 2.

**TABLE 2:** Primers used for PCR

Gene	Forward (5′‐3′)	Reverse (5′‐3′)
PCNA	CCTGCTGGGATATTAGCTCCA	CAGCGGTAGGTGTCGAAGC
Caspase 9	CTTCGTTTCTGCGAACTAACAGG	GCACCACTGGGGTAAGGTTT
Caspase 3	CATGGAAGCGAATCAATGGACT	CTGTACCAGACCGAGATGTCA
Bcl‐2	GGTGGGGTCATGTGTGTGG	CGGTTCAGGTACTCAGTCATCC
Bax	CCCGAGAGGTCTTTTTCCGAG	CCAGCCCATGATGGTTCTGAT
PI3K	TATTTGGACTTTGCGACAAGACT	TCGAACGTACTGGTCTGGATAG
AKT	AGCGACGTGGCTATTGTGAAG	GCCATCATTCTTGAGGAGGAAGT
β‐actin	CATGTACGTTGCTATCCAGGC	CTCCTTAATGTCACGCACGAT

### 2.7 Western blotting analysis

143B and MNNG cells were treated with different concentrations of PB2 (0, 50, 70 and 90 μmol/L) for 48 hours. Total protein was extracted from cells using RIPA lysis buffer containing a protease inhibitor, the mitochondrial and cytoplasmic proteins were extracted using a cell mitochondrial isolation kit. The protein was quantified by BCA protein assay and separated by SDS‐PAGE (7.5%, 10% and 12.5%) for electrophoresis, then transferred to PVDF membranes. Next, the membranes were blocked with 5% non‐fat milk and then incubated with primary antibodies and second antibodies. The protein expression levels were determined with an Odyssey two‐colour infrared laser imaging system (LI‐COR Biosciences). Quantitative analysis was conducted by Image J software.

### 2.8 Animal experiments

The animal protocol was approved by the Ethics Committee of Shanghai Tongji Hospital, and the handling of mice was according to the guidelines of the National Institutes of Health (NIH). BALB/c nude mice (male, 4‐5 weeks) obtained from Shanghai SLAC Laboratory Animal were used to establish the subcutaneous xenograft tumour model. A total of 10 BALB/c nude mice were maintained in a temperature‐controlled room on a 12‐hour light/dark cycle with ad libitum access to food and water. 143B cells were collected and re‐suspended in serum‐free DMEM (5 × 10^6^/mL), and then injected into the right upper flank region of mice (200 μL each). The mice weight and tumour volume were measured every 3 days using the following formula: tumour volume (mm^3^) = length × (width^2^/2). When the subcutaneous tumour volume reached 100 mm^3^, all the mice were randomly assigned into either an experimental group that received PB2 treatment (100 mg/kg, intragastrical gavage) or a normal control (NC) group that received the same volume of saline, once a day for 3 weeks. After the last treatment of PB2 or saline, the mice were sacrificed by cervical dislocation after intraperitoneal injection of 1.25% pentobarbital (40 mg/kg). The xenograft tumours and major organs, including liver, lung, kidney, and heart were harvested from the mice in both groups and immersed in 4% PFA for immunohistochemistry staining.

### 2.9 Immunohistochemistry staining

Haematoxylin and eosin (H&E) staining, Ki‐67 staining, and the TdT‐UTP nick end labelling (TUNEL) staining were carried out, and the staining processes were performed according to the standard protocols. PFA‐immersed tumours were embedded in paraffin and cut into 4 μm‐thick sections. For H&E staining, slides were stained with haematoxylin for 10 minutes and eosin for 5 minutes to visualize the tissue injuries. For Ki‐67 staining, slides were deparaffinized and rehydrated. Antigen retrieval was performed using microwave for 5 minutes, and slices were incubated with 3% hydrogen peroxide for 10 minutes. After that, the slices were incubated with anti‐Ki‐67 antibody overnight at 4°C with gentle shaking and then incubated with secondary antibody at room temperature for 1 hour. For TUNEL assays, sections were dehydrated with ethanol after being deparaffinized twice in xylene, digested with 20 μg/mL proteinase K without DNase at room temperature for 15‐30 minutes and then incubated in the TUNEL reaction mixture at room temperature for 1 hour. Finally, sections were mounted on microscope slides with a coverslip and observed using a microscope (Leica). The brown‐stained cells were regarded as Ki‐67 and TUNEL‐positive cells.

### 2.10 Statistical analysis

Data were expressed as mean ± standard deviation (SD). Group differences were compared by Student's *t* test or one‐way analysis of variance (ANOVA). A two‐way ANOVA with repeated measurements was used to analyse the differences of tumour volume changes between mice in the treated group and untreated group at the different time points. GraphPad Prism 6 software (GraphPad software) was used to analyse the data, and *P* values less than .05 were considered statistically significant.

## 3 RESULTS

### 3.1 PB2 inhibits the proliferation of OS cells

**FIGURE 1:**
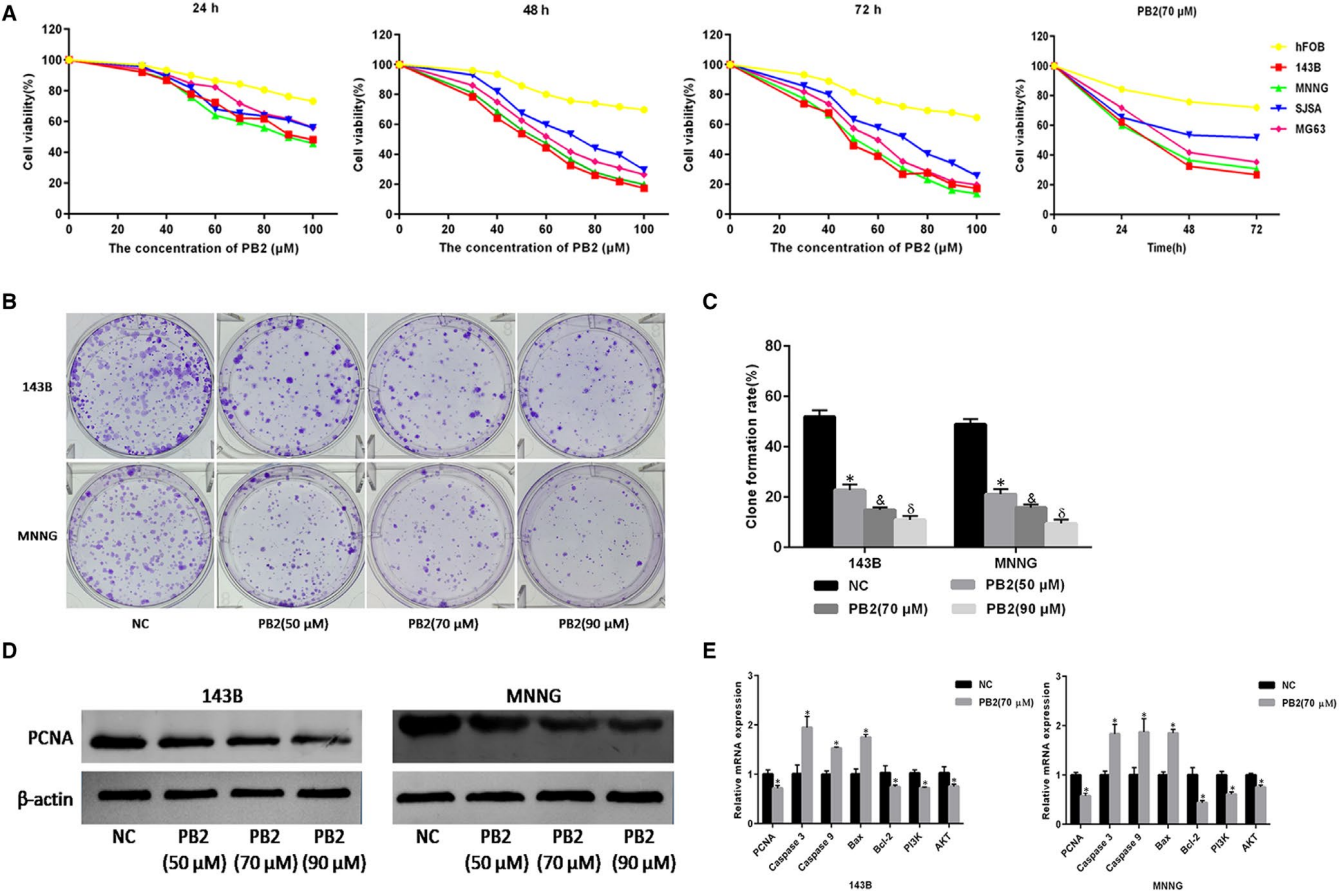
PB2 inhibits OS cell proliferation and induces apoptosis. A, OS cell lines (143B, MNNG, SJSA and MG‐63) and normal human osteoblast cells (hFOB1.19) were treated with PB2 (30‐100 μmol/L) for 24, 48 or 72 h, and cell proliferation was determined using CCK‐8 assays. B,C, Macrograph of colony formation for 143B and MNNG cells (n = 3, ^*,&,δ^ *P* < .001 for PB2 vs NC). D, PCNA protein levels assessed by Western blotting. E, The mRNA expression levels for PCNA, Caspase‐3, Caspase‐9, Bax, Bcl‐2, PI3K and AKT determined by qRT‐PCR (n = 3, ^*^ *P* < .05 for PB2 vs NC)

To investigate the anti‐proliferative effects of PB2, OS cell lines (143B, MNNG, SJSA, and MG‐63) and osteoblast cells (hFOB1.19) were incubated and treated with PB2 in a series of concentrations (30‐100 μmol/L) for 24, 48, and 72 hours. The CCK‐8 assay was used to measure the influence of PB2 on cell proliferation, and the growth curves were plotted. As shown in Figure 1A and Figure S1A‐D, the OS cell viability was decreased after PB2 treatment at different time points and concentrations as compared to the untreated group (*P < *.05), which meant that PB2 could inhibit the growth of OS cells in a dose‐ and time‐dependent manner. In contrast, the osteoblast cell line was less affected over a certain concentration range (Figure S1E). These results suggested that PB2 was able to inhibit the proliferation of OS cells with slightly interfering with the growth of normal osteoblast cells. In addition, we further compared the effect of PB2 (70 μmol/L) on the inhibition of proliferation between OS cells and osteoblast cells at the time point of 48 hours and the results supported that PB2 could specifically decrease the cell viability of tumour cells (*P* < .05, Figure S1F). Finally, we calculated the half‐maximal inhibitory concentration (IC50) at 48 hours for each cell line, respectively. 143B and MNNG cell lines were specifically chosen for the subsequent experiments because these two cell lines were relatively more sensitive to PB2 according to the IC50 (Table 3).

**TABLE 3:** The half‐maximal inhibitory concentration of PB2 at 48 h on cells

Cell lines	143B	MNNG	SJSA	MG‐63	hFOB1.19
IC50 (μmol/L)	57.5	55	77	66	134

Moreover, in order to further verify the role of PB2 in the proliferation of OS cells, colony formation assays and proliferating cell nuclear antigen (PCNA), an indicator reflected the cell proliferation, were employed. Results in Figure 1B,C showed that the clone formation rate was significantly decreased from 51.9% (untreated group) to 22.8%, 14.9%, 10.9% in the 143B group (*P < *.001) and from 48.9% (untreated group) to 21.1%, 15.8%, 9.5% in the MNNG group (*P < *.001) after PB2 treatment at the concentrations of 50, 70 and 90 μmol/L, respectively, for 7 days, which meant that PB2 could inhibit the proliferation and colony formation ability of 143B and MNNG cell lines. The gene expression level and the protein abundance of PCNA measured by qRT‐PCR and Western blotting consistently demonstrated that PB2 inhibited 143B and MNNG cell growth at 48 hours (Figure 1D,E). Collectively, these results confirmed that PB2 could inhibit the proliferation of OS cells.

### 3.2 PB2 induces the apoptosis of OS cells

**FIGURE 2:**
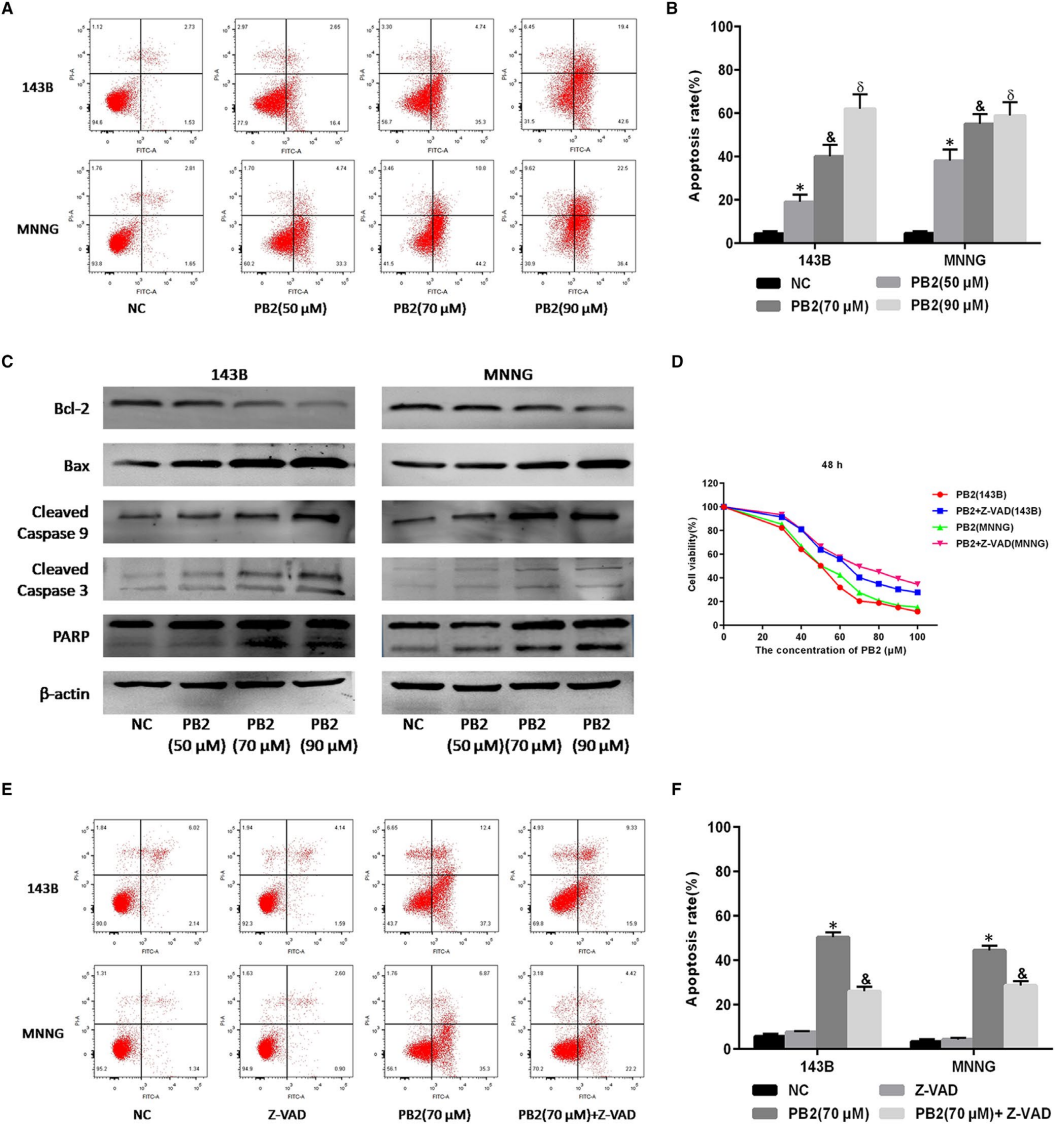
Effects of PB2 on 143B and MNNG cell apoptosis. A,B, 143B and MNNG cells were treated with different concentrations of PB2 (0, 50, 70 and 90 μmol/L) for 48 h. Apoptosis of 143B and MNNG cells was determined by flow cytometry (n = 3, ^*,&,δ^ *P* < .001 for PB2 vs NC). C, Protein levels for Bcl‐2, Bax, cleaved Caspase‐9, cleaved Caspase‐3 and PARP assessed by Western blotting. D, 143B and MNNG cells were treated with PB2 or PB2 + Z‐VAD (50 μmol/L) for 48 h, and cell proliferation was determined by CCK‐8 assays. E,F, Apoptosis of 143B and MNNG cells determined by flow cytometry (n = 3, ^*^ *P < *.001 for PB2 vs NC; ^&^ *P* < 0.001 for PB2 vs PB2 + Z‐VAD)

Whether PB2 could induce the apoptosis of OS cells was determined by flow cytometry and Western blotting. We used different concentrations of PB2 (50, 70 and 90 μmol/L) to treat 143B and MNNG cell lines, respectively. Results of the flow cytometry demonstrated that the OS cells apoptosis rate was significantly increased from 4.3% (untreated group) to 19.1%, 40.0%, 62.0% in the 143B group (*P < *.001) and from 4.5% (untreated group) to 38.0%, 55.0%, 58.9% in the MNNG group (*P* < .001) after the treatment of PB2 at the concentrations of 50, 70 and 90 μmol/L, respectively (Figure 2A,B). Meanwhile, results from Western blotting (Figure 2C) and qRT‐PCR (Figure 1E) showed an increased protein abundance of Bcl‐2‐associated X (Bax), cleaved Caspase‐3, cleaved Caspase‐9 and poly ADP ribose polymerase (PARP), and a decreased protein abundance of B‐cell lymphoma 2 (Bcl‐2) in 143B and MNNG cell lines. The current findings indicated that PB2‐induced apoptosis of OS cells had concentration‐dependent characteristics.

Previous studies have shown that endogenous apoptosis was regulated either in Caspase‐dependent or non‐Caspase‐dependent pathways.^29^ However, whether PB2 induced OS cells apoptosis through Caspase‐independent pathways remained to be determined. Z‐VAD‐FMK is a cell‐permeable and irreversible broad‐spectrum Caspase inhibitor which has been proved able to block all Caspase‐induced apoptosis.^30^ Therefore, we initially treated 143B and MNNG cell lines with Z‐VAD‐FMK at a certain concentration of 50 μmol/L for 1 hour and then treated with different concentrations of PB2. Results from CCK‐8 indicated that Z‐VAD‐FMK could partially prevent the OS cell apoptosis induced by PB2 (Figure 2D). Consistently, the results of flow cytometry assays showed that the OS cells apoptosis rate was 49.7% in the 143B group and 42.2% in the MNNG group after the treatment of PB2 (70 μmol/L), while the apoptosis rate significantly decreased to 25.2% in the 143B group (*P < *.001) and 26.6% in the MNNG group (*P < *.001), respectively, after the combined treatment with PB2 and Z‐VAD‐FMK (Figure 2E,F). These results demonstrated that PB2‐induced OS cell apoptosis was not completely dependent on Caspase enzymes. We speculated that the effects of PB2 on OS cell apoptosis might associate with the apoptosis‐inducing factor (AIF) released by mitochondria, pending further confirmative studies.

### 3.3 PB2 stimulates the release of mitochondrial apoptogenic factors by disrupting mitochondrial membrane permeability

**FIGURE 3:**
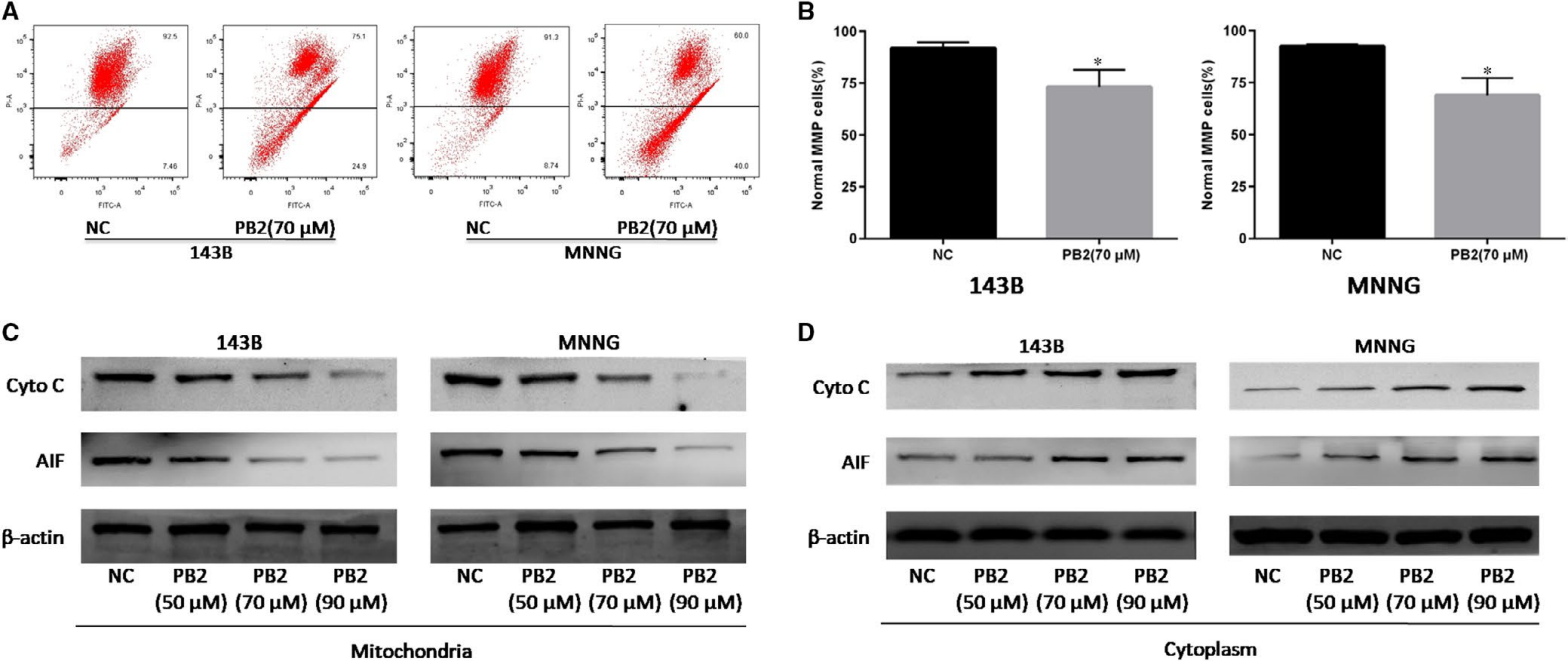
Effects of PB2 on mitochondrial membrane permeability in OS cells. A,B, The MMP of 143B and MNNG cells with or without PB2 treatments determined by JC‐1 using flow cytometry (n = 3, ^*^ *P* < .05 for PB2 vs NC). C,D, Expression of cyto‐C and AIF in mitochondria and cytoplasm of 143B and MNNG cells was determined by Western blotting

The disturbance of mitochondrial membrane potential (MMP, ΔΨm) is an early sign of apoptosis, and the MMP changes induced by apoptotic signals affect the permeability of the mitochondrial membrane.^31^ Increased permeability of the mitochondrial membrane would result in the release of mitochondrial apoptogenic factors, such as cytochrome C (cyto‐C) and AIF from the mitochondrial into the cytoplasm, and lead to the activation of a cascade of apoptotic enzymes.^32^ Therefore, the JC‐1 mitochondrial membrane potential detection kit was used in this study to measure the MMP changes. The results of flow cytometry showed that the percentage of OS cells with normal MMP was significantly decreased from 92.5% to 75.1% in the 143B group (*P* = .020) and from 91.3% to 60.0% in the MNNG group (*P* = .008), respectively, after PB2 treatment at the concentration of 70 μmol/L for 48 hours. These results indicated that PB2 might promote apoptosis of OS cells by disturbing the MMP and breaking the permeability of the mitochondrial membrane (Figure 3A,B). We then separated the mitochondrial from the cytoplasm, measured the changes of cyto‐C and AIF in 143B and MNNG cell lines, which had been treated with different concentrations of PB2. The results of Western blotting suggested that the cyto‐C and AIF levels were significantly reduced in the mitochondrial, but just the opposite, that were increased in the cytoplasm (Figure 3C,D). These results further indicated that PB2‐induced OS cell apoptosis might attribute to the disruption of mitochondrial membrane permeability, and leading to the release of cyto‐C and AIF from the mitochondrial into the cytoplasm.

### 3.4 PB2 induces OS cell apoptosis by suppressing the PI3K/AKT signalling pathway

**FIGURE 4:**
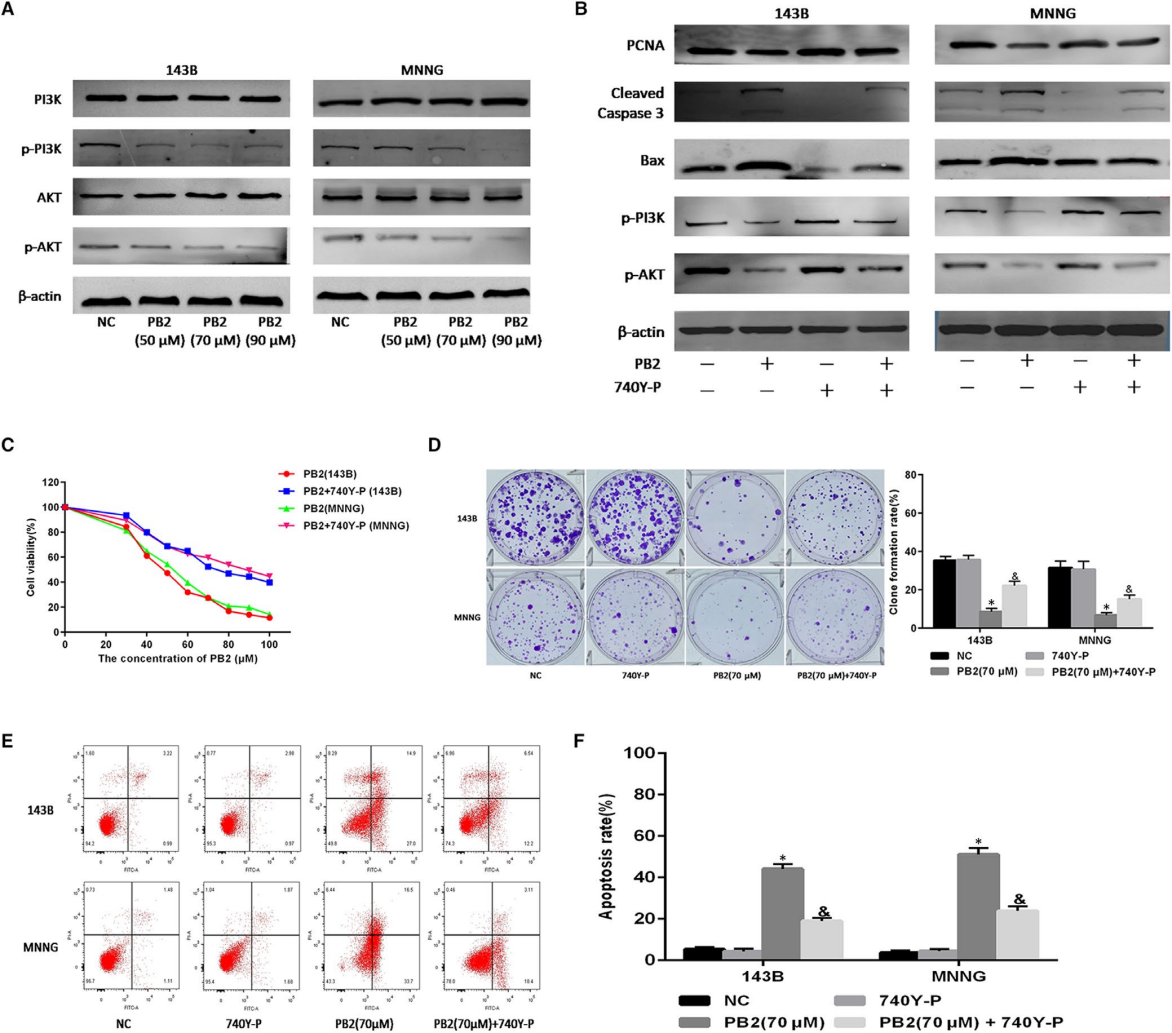
The role of the PI3K/AKT pathway in OS cell proliferation and apoptosis. A, Expressions of PI3K, p‐PI3K, AKT and p‐AKT in 143B and MNNG cells determined by Western blotting. B, 143B and MNNG cells treated with PB2 or PB2 + 740Y‐P (20 μmol/L) for 48 h, and expression of PCNA, cleaved Caspase‐3, Bax, p‐PI3K and p‐AKT determined by Western blotting. C, Cell proliferation determined using the CCK‐8 assays. D, Macrograph of colony formation in 143B and MNNG cells (n = 3, ^*^ *P* < .05 for PB2 vs NC; ^&^ *P* < .05 for PB2 vs PB2 + 740Y‐P). E,F, Apoptosis of 143B and MNNG cells determined by flow cytometry (n = 3, ^*^ *P* < .05 for PB2 vs NC; ^&^ *P* < .05 for PB2 vs PB2 + 740Y‐P)

The PI3K/AKT signalling pathway is a major intracellular signalling cascade, regulating tumour cell proliferation, apoptosis, and migration.^33^ Previous studies have reported that aberrant activation of the PI3K/AKT pathway was closely associated with the negative regulation of tumour cell apoptosis.^34^ Therefore, we further analysed whether PB2‐induced apoptosis of OS cells was through suppressing the PI3K/AKT signalling pathway. The qRT‐PCR and Western blotting results indicated that mRNA levels of PI3K and AKT were significantly decreased (Figure 1E), and similarly, protein levels of p‐PI3K and p‐AKT were significantly reduced in 143B and MNNG cell lines after the treatment with different concentrations of PB2 (Figure 4A). Taken together, these results demonstrated that PB2 could induce OS cell apoptosis through inactivating the PI3K/AKT signalling pathway.

### 3.5 Activation of the PI3K/AKT pathway using 740Y‐P reverses the effects of PB2 on OS cell proliferation and apoptosis

To further investigate whether the antitumoral effects of PB2 were mediated by inhibiting of the PI3K/AKT signalling pathway, 143B and MNNG cell lines were treated with different concentrations of PB2, combined with or without 740Y‐P (a PI3K agonist) for 48 hours. The Western blotting analysis demonstrated that 740Y‐P could reverse the changes in the abundance of p‐PI3K, p‐Akt, Bax, PCNA and cleaved Caspase‐3 proteins in 143B and MNNG cell lines induced by PB2 (Figure 4B). Moreover, we found that 740Y‐P could partially reverse the inhibitory effects of PB2 on OS cell proliferation measured by CCK‐8 and colony formation assays (Figure 4C,D). Specifically, the clone formation rate was 8.6% in the 143B group and 6.8% in the MNNG group after the treatment of PB2 (70 μmol/L), while the clone formation rate was significantly increased to 22.2% in the 143B group (*P* = .001) and 15.2% in the MNNG group (*P* = .004), respectively, after the combined treatment of PB2 (70 μmol/L) and 740Y‐P. In addition, the results of flow cytometer analysis demonstrated that 740Y‐P could simultaneously decrease the apoptosis rates of OS cells (Figure 4E,F). Specifically, the OS cell apoptosis rate was 41.9% in the 143B group and 50.2% in the MNNG group after the treatment of PB2 (70 μmol/L). While the cell apoptosis rate was significantly decreased to 18.7% in the 143B group (*P* < .001) and 21.5% in the MNNG group (*P* < .001), respectively, after the combined treatment of PB2 (70 μmol/L) and 740Y‐P. On the whole, these results confirmed that PB2 inhibited proliferation and induced apoptosis in OS cells by suppressing the PI3K/AKT signalling pathway.

### 3.6 PB2 inhibits the growth of OS xenografts in vivo

**FIGURE 5:**
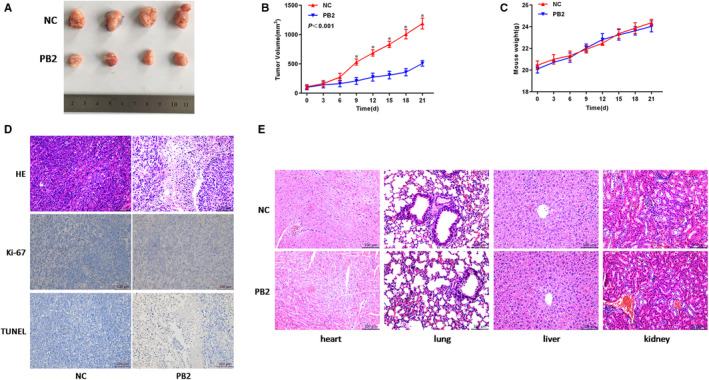
Effects of PB2 on OS tumour growth in xenograft mice model. A, Gross observation of 143B xenograft tumours harvested from the nude mice. B, Changes in tumour volumes recorded at different time points (n = 5, ^*^ *P < *.001). C, Effects of PB2 on the bodyweight of mice. D, The proliferation and apoptosis of tumour tissues with or without PB2 treatments indicated by H&E, Ki‐67 and TUNEL staining. E, No significant pathological changes observed in major organs including heart, lung, liver, and kidney demonstrated by H&E staining (magnification 200×)

In addition to the findings from in vitro experiments, we also conducted in vivo experiments by establishing a xenograft model to further determine whether PB2 could effectively inhibit the OS cell growth. As shown in Figure 5A, the growths of xenograft tumours harvested from the PB2‐treated group were remarkably inhibited as compared to the NC group. Specifically, the tumour growth rate in the PB2‐treated group was significantly slower as compared to that in the NC group (*P* < .001, Figure 5B), while the bodyweights of mice in both groups were comparative (Figure 5C). We then further investigated the underlying pathological changes by immunohistochemistry staining (Figure 5D). We observed that there were more necrotic lesions in the tumour tissues in the PB2‐treated group, while numerous tumour cells existed in the NC group by H&E staining. In consistence, there were fewer proliferative cells and more apoptotic cells in the PB2‐treated group than that in the NC group revealed by Ki‐67 and TUNEL assays. Finally, we assessed the effects of PB2 on major organs including liver, heart, lung, and kidney, and the results from H&E staining showed that PB2 exerted no significant toxicity on these normal organs (Figure 5E). Overall, these findings confirmed that PB2 suppressed the growth of OS xenografts in vivo by inhibiting OS cell proliferation and inducing apoptosis.

## 4 DISCUSSION

Current therapeutic strategies for OS include surgical resection and chemotherapy.^6^, ^35^ There exist challenges of performing tumour resection surgery for young children with an immature skeleton, and the selection of surgical procedures should consider the tumour location and metastasis, as well as the neurovascular invasion and functional disability.^36^, ^37^, ^38^ As for chemotherapy, it has been applied pre‐ and post‐surgery to offer an opportunity for well planning the surgery or to modify the clinical outcomes.^39^, ^40^, ^41^ But acute and/or chronic toxicities might associate with chemotherapy for the treatment of OS.^8^, ^9^ Thus, it is of great significance to develop novel and efficient chemotherapeutic drugs. PB2 has been evidenced to possess anti‐tumour properties.^20^, ^23^, ^28^ However, whether PB2 could be used for OS treatment remains to be determined. In the current studies, we thus conducted both in vitro and in vivo experiments to systematically investigate the effects of PB2 on OS cell proliferation and apoptosis, and also explored the potential molecular mechanisms.

The proliferation and differentiation of cancer cells are important for tumour invasion.^42^ Therefore, inhibition of proliferation and promotion of apoptosis in OS cells are critical for OS treatments. In the present study, we found that PB2 could significantly inhibit OS cell (143B and MNNG cell lines) proliferation in a time‐ and dose‐dependent manner. It is worth mentioning that there were little toxic effects of PB2 on normal human osteoblast cells, which indicated that PB2 might be a relatively safe natural drug for the treatment of OS. Moreover, we also found that PB2 treatment could induce OS cell apoptosis in a dose‐dependent manner. Results from the in vivo experiments further confirmed that PB2 could not only inhibit the OS cell proliferation and thus reduce the xenograft tumour volumes, but also promote the apoptosis reflected by an increased number of TUNEL‐positive cells in tumour tissues harvested from the xenograft mice. In summary, these results demonstrated that PB2 exerted significant anti‐proliferation effects and was able to efficiently induce apoptosis in OS cells, both in vitro and in vivo.

Apoptosis, comprising both intrinsic and extrinsic pathways, plays an important role in maintaining the balance between cell death and growth.^43^ Disruption of MMP and the release of mitochondrial apoptogenic factors are key determinants of the mitochondrial‐mediated intrinsic apoptotic pathway.^44^ BCL‐2 family proteins are key regulators of MMP, and both anti‐apoptotic and pro‐apoptotic members have been identified.^31^ The balance between these two groups of BCL‐2 proteins can largely determine cell fate decisions between life and death. Pro‐apoptotic activator proteins can be bound and sequestered by anti‐apoptotic proteins such as BCL‐2 and Bcl‐X_L_ under physiological conditions.^31^ However, when these anti‐apoptotic proteins are saturated or absent under pathological conditions, pro‐apoptotic activator proteins can activate Bax and Bak, and cause them to oligomerize and form macropores at the mitochondrial surface, leading to the increase of mitochondrial membrane permeability and the release of mitochondrial apoptogenic molecules such as cyto‐C and AIF into the cytoplasm. The released apoptogenic molecules promoted the activation of the initiator Caspase‐9 and the executioner Caspase‐3, which induced the cells apoptosis.^31^ In this study, we found that the Bcl‐2 and the mitochondrial cyto‐C proteins were decreased following the PB2 treatments, but in contrast, the levels of Bax, cleaved Caspase‐3, cleaved Caspase‐9 and the cytoplasm cyto‐C protein were increased. Additionally, damage to mitochondrial function also contributed to AIF transferring from the mitochondrial to the cytoplasm, resulting in nuclear DNA agglutination. Taken together, these results indicated that PB2 could disrupt the mitochondrial outer membrane permeability and thus initiate the intrinsic apoptotic pathway.

The PI3K/AKT pathway is an important intracellular signalling pathway that plays an indispensable role in the regulation of various cell biological behaviours, such as cell proliferation and apoptosis.^33^, ^45^, ^46^ Most studies have indicated that the PI3K/AKT pathway is active in osteosarcoma cells, and its inhibition can reduce OS cell proliferation, invasion and induce apoptosis.^15^, ^47^ BCL‐2 family proteins are key downstream targets of the PI3K/AKT pathway and are mitochondrial outer membrane proteins able to regulate mitochondrial‐mediated apoptosis.^31^, ^47^ Activated PI3K/AKT can decrease the levels of pro‐apoptotic proteins, and increase the levels of anti‐apoptotic proteins, which can block the release of cyto‐C from mitochondria and prevent the activation of Caspase apoptosis proteins. Therefore, the PI3K/AKT signalling pathway has the potential for therapeutic intervention against OS.

**FIGURE 6:**
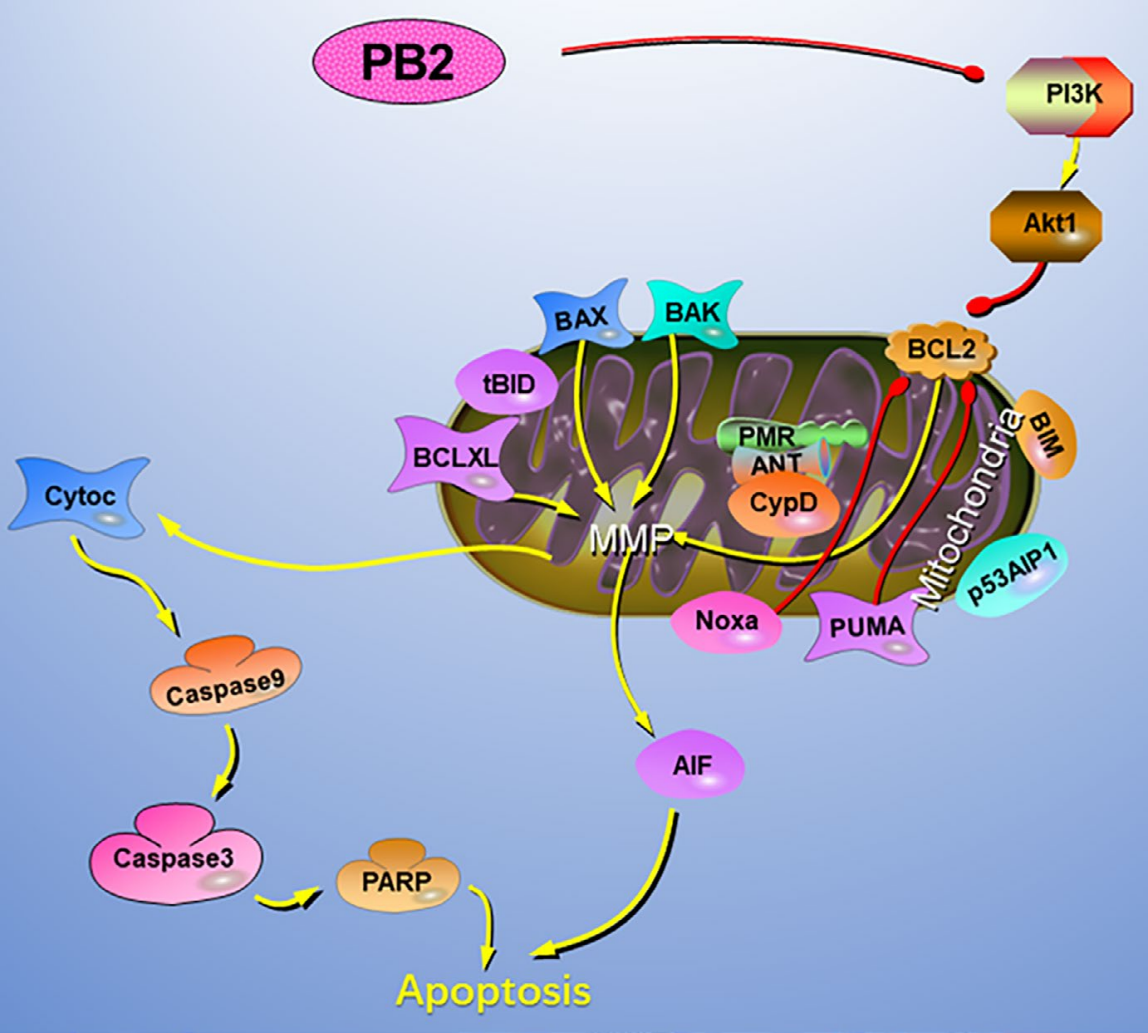
The potential mechanisms of PB2‐induced apoptosis in OS cells. PB2 affects the balance of anti‐apoptotic and pro‐apoptotic proteins through suppressing the PI3K/AKT signalling pathway. Moreover, PB2 increases the mitochondrial membrane permeability and causes the release of cyto‐C and AIF from mitochondrial into the cytoplasm, resulting in the activation of intrinsic apoptotic pathways and nuclear DNA agglutination

Findings from this study suggested that PB2 could reduce the expression of PI3K and AKT at both mRNA and protein levels, which implied that PB2 could inhibit the PI3K/AKT signalling pathway. In addition, activating the PI3K/AKT signalling pathway by 740Y‐P could reverse the effects of PB2 on the proliferation and apoptosis of OS cells. Therefore, these results demonstrated that PB2 inhibited proliferation and induced apoptosis in OS cells through suppression of the PI3K/AKT signalling pathway (Figure 6).

The current studies have several limitations. First, we only explored the inhibitory effect of PB2 on the growth of subcutaneously implanted tumours in mice within three weeks, but did not explore whether PB2 could prolong the median survival time or decrease the risk of developing tumours in a relatively long time. Nevertheless, findings from the present studies would help to set up a system for the future studies to further determine the effects of PB2 on the long‐term prognosis and tumorigenesis. Second, we have not carried out clinical trials to explore the clinical efficacy of PB2 in the treatment of osteosarcoma patients at present. One of the challenges related to employing PB2 as anticancer agents is the poor bioavailability, which might associate with its low water solubility, instability under the physicochemical conditions of the gastrointestinal tract, as well as the difficulty of cell membrane diffusion.^48^, ^49^, ^50^, ^51^ However, some promising approaches have been proposed to enhance the bioavailability of polyphenol, mainly including (a) the structural modification, such as methylation, hydroxylethylation and amino acid substitution, to decrease the physiochemical degradation and enhance the intestinal absorption rate,^52^, ^53^ (b) bioactive carrier systems, such as phytosome/liposome vehicles, solid lipid nanoparticles, microencapsulation and nano‐graphene oxide systems, to deliver the agents to the target sites with minimal side effects,^48^, ^54^ (c) co‐administration with other dietary components, such as intestinal absorption enhancers and dietary lipids, to improve the permeability of intestinal epithelial cells, delay gastric empty and prolong bowel transit time.^54^, ^55^ We will conduct clinical studies to further investigate whether PB2 assisted with nanomaterials could facilitate its bioavailability, and thus improve its therapeutic effects on osteosarcoma patients in the future.

In conclusion, our findings suggested that PB2 can suppress proliferation and induce apoptosis in 143B and MNNG cells via inactivating the PI3K/AKT signalling pathway. The antitumoral activities of PB2 may provide a new chemotherapeutic route for the treatment of OS in future clinic.

## CONFLICT OF INTEREST

The authors declare that they have no conflicts of interest.

## AUTHOR CONTRIBUTIONS

**Xinbo Wu:** Data curation (lead); investigation (lead); project administration (lead); writing‐original draft (lead). **Haiyang Yu:** Data curation (equal); formal analysis (lead); investigation (supporting); project administration (supporting); writing‐original draft (equal). **Haichao Zhou:** Investigation (supporting); project administration (supporting). **Zihua Li:** Formal analysis (supporting); investigation (supporting); project administration (supporting). **Hui Huang:** Data curation (supporting); project administration (supporting). **Fajiao Xiao:** Formal analysis (supporting); investigation (supporting); project administration (supporting). **Shaochen Xu:** Data curation (supporting); investigation (supporting). **Yunfeng Yang:** Conceptualization (lead); supervision (lead); validation (equal); Writing‐review & editing (lead).

## Supporting information

Fig S1

## Data Availability

The data that support the findings of this study are available from the corresponding author on reasonable request.
