# Supplementary figures and images for: Proanthocyanidin B2 inhibits proliferation and induces apoptosis of osteosarcoma cells by suppressing the PI3K/AKT pathway
Source: J Cell Mol Med. 2020 Sep 10;24(20):11960–71. doi: 10.1111/jcmm.15818 (PMC7579710; doi:10.1111/jcmm.15818)

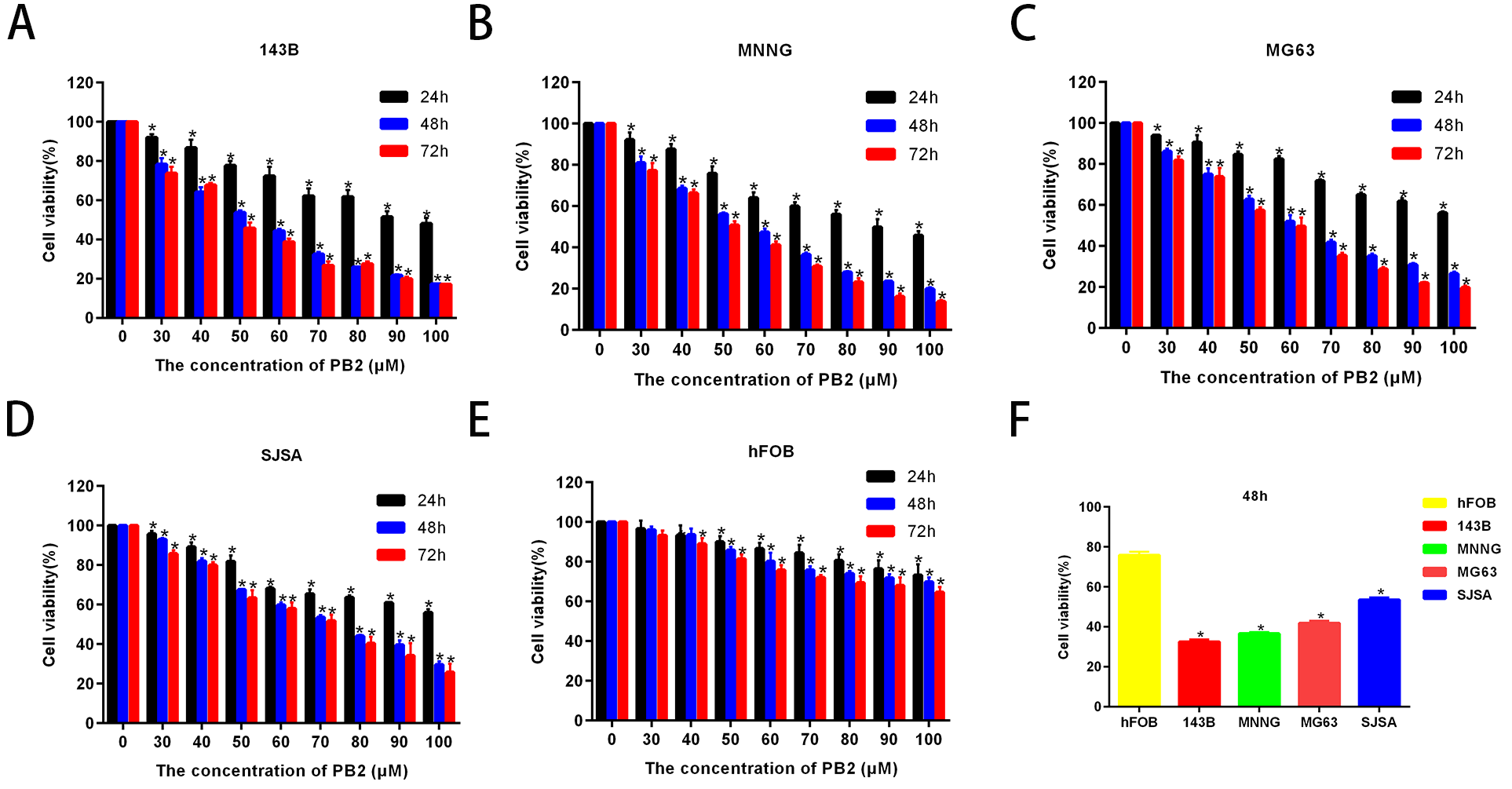

Supplement: Supplementary file 1 — Fig S1 [file JCMM-24-11960-s001.tif]
